# Treatment of US Children With Attention-Deficit/Hyperactivity Disorder in the Adolescent Brain Cognitive Development Study

**DOI:** 10.1001/jamanetworkopen.2023.10999

**Published:** 2023-04-28

**Authors:** Mark Olfson, Melanie M. Wall, Shuai Wang, Gonzalo Laje, Carlos Blanco

**Affiliations:** 1 Department of Psychiatry, College of Physicians and Surgeons, Columbia University and the New York State Psychiatric Institute, New York; 2 Division of Epidemiology, Services, and Prevention Research, National Institute on Drug Abuse, Rockville, Maryland; 3 Maryland Institute for Neuroscience and Development Inc, Chevy Chase; 4 Washington Behavioral Medicine Associates, LLC, Chevy Chase, Maryland; 5 Department of Psychiatry, Texas Tech University Health Science Center, Lubbock

## Abstract

**Importance:**

Characterizing the extent and pattern of unmet needs for treatment of children with attention-deficit/hyperactivity disorder (ADHD) could help target efforts to improve access to ADHD medications and outpatient mental health care.

**Objective:**

To describe current ADHD medication use and lifetime outpatient mental health care among a large national sample of children with ADHD.

**Design, Setting, and Participants:**

This study uses cross-sectional survey data from the first wave of the Adolescent Brain and Cognitive Development Study (n = 11 723), conducted from June 1, 2016, to October 15, 2018, among 1206 school children aged 9 and 10 years who met parent-reported *Diagnostic and Statistical Manual of Mental Disorders* (Fifth Edition) criteria for current ADHD. Statistical analysis was performed from March 23, 2022, to March 10, 2023.

**Main Outcomes and Measures:**

Current ADHD medications including stimulants and nonstimulants, lifetime outpatient mental health care, or either treatment. Weighted results are reported.

**Results:**

Among a sample of 11 723 children, 1206 had parent-reported ADHD (aged 9-10 years; 826 boys [68.2%]; 759 White, non-Hispanic children [62.2%]), 149 (12.9%) were currently receiving ADHD medications. Children receiving ADHD medications included a significantly higher percentage of boys (15.7% [121 of 826]) than girls (7.0% [28 of 108]), White children (14.8% [104 of 759]) than Black children (9.4% [22 of 206]), children of parents without a high school education (32.2% [9 of 36]) than of parents with a bachelor’s degree or higher (11.5% [84 of 715]), and children with the combined subtype of ADHD (17.0% [83 of 505]) than with the inattentive subtype (9.5% [49 of 523]). Approximately 26.2% of children (301 of 1206) with parent-reported ADHD had ever received outpatient mental health care. Children receiving outpatient mental health care included a significantly higher percentage of children whose parents had a high school education (36.2% [29 of 90]) or some college (31.0% [109 of 364]) than a bachelor’s degree or higher (21.3% [153 of 715]), children with family incomes of less than $25 000 (36.5% [66 of 176]) or $25 000 to $49 999 (27.7% [47 of 174]) than $75 000 or more (20.1% [125 of 599]), and children with the combined subtype of ADHD (33.6% [166 of 505]) than with the predominantly inattentive subtype (20.0% [101 of 523]) or the hyperactive-impulsive subtype (22.4% [34 of 178]) of ADHD.

**Conclusions and Relevance:**

This cross-sectional study of children with parent-reported ADHD suggests that most were not receiving ADHD medications and had never received outpatient mental health care. Gaps in treatment, which were not directly associated with socioeconomic disadvantage, underscore the challenges of improving communication and access to outpatient mental health care for children with ADHD.

## Key Points

**Question:**

What percentage of children with parent-reported attention-deficit/hyperactivity disorder (ADHD) receive treatment?

**Findings:**

In this cross-sectional study of 11 723 children aged 9 and 10 years, 12.9% of children with parent-reported ADHD were currently receiving ADHD medications, including 15.7% of boys, 7.0% of girls, 14.8% of White children, 9.4% of Black children, 32.3% of children of parents without a high school education, and 11.5% of children whose parents had a bachelor’s degree. Approximately 26.2% of children with parent-reported ADHD had ever received outpatient mental health care.

**Meaning:**

This study suggests that pervasive gaps exist in the treatment of children, especially girls, with parent-reported ADHD.

## Teaser

This cross-sectional study uses data from the Adolescent Brain and Cognitive Development Study to describe current attention-deficit/hyperactivity disorder (ADHD) medication use and lifetime outpatient mental health care among a large national sample of children with ADHD.

## Introduction

In the US and other high-income countries, an increasing number of children receive a clinical diagnosis of and are treated for attention deficit/hyperactivity disorder (ADHD).^1,2,3,4,5,6,7^ The percentage of US children whose parents report their child has received a diagnosis of ADHD increased from 5.5% in 1999 to 9.8% in 2018.^8^ The Centers for Disease Control and Prevention (CDC) further report that 77% of US children who received a diagnosis of ADHD received treatment and that 69% received medications.^8,9^

Reports of increasing rates of children treated with medications for ADHD have fueled public^10,11,12^ and professional^13^ perceptions that childhood ADHD is overdiagnosed and overtreated. However, a focus on the increasing numbers of children treated for ADHD does not give a sense of what fraction of children in the population with ADHD receive treatment. To estimate the unmet need for ADHD treatment, it is necessary to ascertain children from outside of clinical settings, evaluate them for ADHD, and assess their treatment use.

Beyond estimating the overall proportion of children with ADHD who receive treatment, it is important to understand which children with ADHD are more or less likely to receive treatment. Characterizing treatment patterns could guide efforts to improve mental health care access for underserved groups. Prior studies have reported that, among children with ADHD, girls are less likely than boys^2,14,15,16,17^ to receive ADHD medications, as are children from ethnic and racial minority groups compared with non-Hispanic White (hereafter, White) children.^15,16,18,19^ Although some research further suggests that children with ADHD who are from lower-income families compared with higher-income families are less likely to receive ADHD medications,^19,20,21,22^ other studies have reported the reverse.^9,23,24^ In a school-based sample, children with the combined subtype of ADHD were more likely than those with either the inattentive or hyperactive-impulsive subtype of ADHD to receive stimulants.^25^ One study found that hyperactive symptoms were associated with ADHD medication use,^26^ while another found that severity of inattention and hyperactivity-impulsivity symptoms had similar correlations with ADHD medication treatment.^27^

We characterize treatment patterns among a cohort of US school children aged 9 and 10 years whose parents reported that their children met *Diagnostic and Statistical Manual of Mental Disorders* (Fifth Edition) (*DSM-5*) ADHD criteria. We estimate the percentages of these children who were currently treated with ADHD medications, had ever received outpatient mental health care, or both. We hypothesized that most children with parent-reported ADHD would not be currently receiving ADHD medications or have never received outpatient mental health care and that treatment gaps would be greater for girls than boys and for children from families of lower socioeconomic status.

## Methods

### Data Source

Analyses were performed with baseline data from the Adolescent Brain Cognitive Development (ABCD) Study of 11 723 children ages 9 and 10 recruited from primary schools and public school systems at 21 sites nationwide from June 1, 2016, to October 15, 2018. By recruiting from demographically, geographically, and socioeconomically diverse school systems, the ABCD Study sought a sample reflecting the US demographic composition.^28^ A stratified probability sample of schools was selected from each site to approximate national representativeness with respect to sex, race and ethnicity, and socioeconomic status. Survey weights were developed to further balance the sample with respect to basic sociodemographic characteristics, including child age, sex, and race and ethnicity, as well as household family income, family type (married parents or single parent), household size, parent’s work status, and US Census region.^29^ Parent and child recruitment within schools was voluntary and therefore self-selected, and response rates were not available to incorporate into the sampling weights. As a consequence, the sample is not representative of the selected school systems or the nation.^30^ The institutional review board of the University of California, San Diego gave centralized approval for data collection, and each individual study site obtained approval from their local institutional review board. All parents and caregivers provided written informed consent, and the children provided verbal assent. This report followed the Strengthening the Reporting of Observational Studies in Epidemiology (STROBE) reporting guideline for reporting cross-sectional studies.

Inclusion criteria were age 9.00 to 10.99 years and attendance at the sampled school. In brief, exclusion criteria included not being fluent in English; neither parent being fluent in English or Spanish; a major medical or neurologic condition; gestational age less than 28 weeks or birth weight less than 1200 g; a contraindication to magnetic resonance image scanning; a history of traumatic brain injury; a diagnosis of moderate or severe autism spectrum disorder, intellectual disability, or schizophrenia; or a substance use disorder.

### Sociodemographic Characteristics

Sociodemographic characteristics including age, birth sex, parent-reported race and ethnicity, parental marital status, and highest parental educational level were derived from the PhenX toolkit.^31^ Family income was assessed with General Social Survey items,^32^ and the language predominantly spoken to the child by their parent or guardian was coded as English vs other.

### ADHD Subtype and Comorbid Mental Health Disorders

Current mental health disorders were assessed with the parent version of the self-administered computerized Kiddie Schedule for Affective Disorders and Schizophrenia for *DSM-5* (KSADS).^33,34^ Following *DSM-5* criteria, respondents were classified by current ADHD symptoms as combined, predominantly inattentive, or predominantly hyperactive or impulsive presentations. Prior research demonstrates 90% agreement between parent and clinician ADHD current diagnoses from the computerized KSADS (Cohen κ = 0.79).^34^

Some analysis involved comparing treatment of children with ADHD with or without the following comorbid conditions: disruptive behavior disorders (oppositional defiant or conduct disorder); anxiety (panic, separation anxiety, social anxiety, and generalized anxiety disorder) or mood (major depressive, persistent depressive, unspecified depressive, and bipolar disorder) disorders; and obsessive-compulsive disorder.

In a secondary analysis (n = 4594), the ADHD definition was narrowed to require parent-reported ADHD and a T score of 65 or more on the Brief Problem Monitor teacher-version attention scale.^35^ Children with psychotic disorders, bipolar disorder, and IQ less than 70 (measured by the Wechsler Intelligence Scale for Children, Fifth Edition)^36^ were also excluded. Requirement of teacher ratings follows the *DSM-5* criteria of symptoms in 2 or more settings.^37^

### Mental Health Treatment

Parents were asked what medications their children had taken in the past 2 weeks. Medications for ADHD included stimulants (amphetamine, dexmethylphenidate, dextroamphetamine, dextroamphetamine-amphetamine, lisdexamfetamine, methamphetamine, and methylphenidate) and nonstimulants (atomoxetine, clonidine, and guanfacine). Parents were also asked whether their child had ever received outpatient mental health care. Three nonmutually exclusive groups were considered: ADHD medications, any outpatient mental health care, or either (“any treatment”).

### Statistical Analysis

Statistical analysis was performed from March 23, 2022, to March 10, 2023. First, the background characteristics of children with or without parent-reported ADHD were evaluated. Second, among children with parent-reported ADHD, the percentages of children with any treatment were determined overall and stratified by sociodemographic and diagnostic characteristics. A series of unadjusted logistic regressions was performed with each treatment measure as dependent variables to generate odds ratios (ORs) with 95% CIs. Corresponding logistic regressions were adjusted for ADHD subtype and comorbid psychiatric disorders. Third, the percentages of children with ADHD currently receiving stimulant medications or other ADHD medications were determined stratified by background characteristics. Fourth, among children who met the more restricted ADHD criteria involving parent and teacher ratings, ADHD treatment rates were determined overall. For these analyses, nonresponse inverse probability weights were created using logistic regression of the Brief Problem Monitor teacher response (present or absent) as the dependent variable associated with child sociodemographic variables (eTable in Supplement 1). Statistical tests were 2-sided. Analyses were performed in SAS, version 9.4 (SAS Institute Inc) and incorporated propensity-based population weights.

## Results

### Background Characteristics

Compared with children without parent-reported ADHD (n = 10 517), children with parent-reported ADHD (n = 1206) included a larger percentage of boys (68.2% [826 of 1206] vs 49.4% [5295 of 10 517]), White children (62.2% [759 of 1206] vs 55.3% [6234 of 10 517]), Black children (15.2% [206 of 1206] vs 13.6% [1614 of 10 517]), and children whose parents had never married (16.8% [175 of 1206] vs 14.2% [1261 of 10 517]) (Table 1). Children with parent-reported ADHD were also more likely than those without parent-reported ADHD to have each of the psychiatric comorbid conditions.

**Table 1.** Sociodemographic Characteristics of Children With and Without Parent-Reported Current ADHD^a^

Characteristic	Children, No. (%)^b^	
	With ADHD (n = 1206)	Without ADHD (n = 10 517)
Sex, child		
Male	826 (68.2)	5295 (49.4)
Female	380 (31.8)	5222 (50.6)
Race and ethnicity, child		
Black, non-Hispanic	206 (15.2)	1614 (13.6)
Hispanic	192 (18.9)	2166 (24.5)
White, non-Hispanic	759 (62.2)	6234 (55.3)
Other, non-Hispanic^c^	25 (3.8)	376 (6.5)
Language spoken at home		
English	1078 (94.3)	8997 (86.6)
Other	58 (5.7)	1054 (13.4)
Marital status, parent		
Married or living with partner	841 (63.3)	7741 (67.8)
Separated, divorced, or widowed	179 (19.9)	1430 (18.0)
Never married	175 (16.8)	1261 (14.2)
Highest parental educational level		
<12th Grade	36 (3.7)	541 (6.3)
High school graduate or GED certification	90 (8.4)	1019 (11.4)
Some college or Associate’s degree	364 (35.6)	2674 (29.1)
Bachelor’s degree or higher	715 (52.3)	6270 (53.1)
Annual family income		
<$25 000	176 (20.3)	1432 (18.7)
$25 000-$49 999	174 (21.8)	1392 (19.9)
$50 000-$74 999	159 (17.2)	1321 (17.5)
≥$75 000	599 (40.7)	5471 (43.8)
Comorbid disorders		
Disruptive behavior disorder	405 (34.3)	477 (4.6)
Anxiety disorder or OCD	193 (17.3)	231 (2.2)
Mood disorder	64 (5.6)	51 (0.5)

Abbreviations: ADHD, attention-deficit/hyperactivity disorder; GED, General Educational Development; OCD, obsessive-compulsive disorder.

^a^ Current ADHD diagnosis was based on parent report. Data were from the first wave of the Adolescent Brain Cognitive Development Study.

^b^ Percentages are weighted.

^c^ Includes Alaska Native, American Indian or Native American, Asian Indian, Chinese, Filipino, Guamanian, Japanese, Korean, Native Hawaiian, Other Pacific Islander, Samoan, Vietnamese, other Asian, and other race.

### Treatment of ADHD by Sociodemographic Characteristics

Approximately 10.1% (n = 1206) of children had parent-reported ADHD. Among this group, 149 (12.9%) were currently receiving ADHD medications, 301 (26.2%) had ever received outpatient mental health care, and 400 (34.8%) had received either (Table 2). Among children without parent-reported ADHD (n = 10 517), 1.9% (n = 180), or 57.0% of all children receiving ADHD medications, were currently receiving ADHD medications.

**Table 2.** Percentages of Children With Parent-Reported Current ADHD Who Received ADHD Treatment, Total and Stratified by Sociodemographic Characteristics^a^

Characteristic	Any treatment^b^		ADHD medications		Outpatient mental health care	
	No. (%)	OR (95% CI)	No. (%)	OR (95% CI)	No. (%)	OR (95% CI)
Total (n = 1206)	400 (34.8)	NA	149 (12.9)	NA	301 (26.2)	NA
Sex, child						
Male (n = 826)	292 (37.1)	1.39 (0.99-1.93)	121 (15.7)	2.46 (1.42-4.27)	211 (26.7)	1.08 (0.72-1.62)
Female (n = 380)	108 (29.8)	1 [Reference]	28 (7.0)	1 [Reference]	90 (25.2)	1 [Reference]
Race and ethnicity, child^c^						
White, non-Hispanic (n = 759)	258 (37.1)	1 [Reference]	104 (14.8)	1 [Reference]	185 (26.9)	1 [Reference]
Hispanic (n = 192)	55 (30.0)	0.73 (0.50-1.05)	18 (10.5)	0.67 (0.43-1.07)	45 (24.4)	0.88 (0.50-1.54)
Black, non-Hispanic (n = 206)	74 (33.9)	0.87 (0.60-1.26)	22 (9.4)	0.60 (0.36-0.99)	61 (28.0)	1.06 (0.69-1.63)
Language spoken at home						
English (n = 1078)	364 (35.3)	1 [Reference]	134 (12.9)	1 [Reference]	272 (26.5)	1 [Reference]
Other (n = 58)	14 (24.2)	0.58 (0.30-1.13)	7 (12.2)	0.94 (0.44-2.01)	9 (14.4)	0.47 (0.23-0.94)
Marital status, parent						
Married or living with partner (n = 841)	258 (31.6)	1 [Reference]	103 (12.8)	1 [Reference]	188 (22.8)	1 [Reference]
Separated, divorced, or widowed (n = 179)	68 (40.6)	1.48 (0.95-2.31)	23 (14.1)	1.11 (0.72-1.73)	56 (33.2)	1.69 (0.98-2.91)
Never married (n = 175)	73 (41.5)	1.54 (0.81-2.90)	23 (12.9)	1.01 (0.54-1.88)	56 (32.0)	1.59 (0.85-2.99)
Highest parental educational level						
<12th Grade (n = 36)	16 (50.1)	2.40 (0.91-6.31)	9 (32.2)	3.66 (1.72-7.77)	10 (28.3)	1.46 (0.72-2.93)
High school graduate or GED certification (n = 90)	34 (40.9)	1.65 (0.95-2.87)	14 (15.3)	1.39 (0.72-2.67)	29 (36.2)	2.10 (1.22-3.62)
Some college or Associate’s degree (n = 364)	139 (39.7)	1.57 (1.11-2.22)	42 (12.6)	1.11 (0.59-2.09)	109 (31.0)	1.66 (1.25-2.20)
Bachelor’s degree or higher (n = 715)	211 (29.5)	1 [Reference]	84 (11.5)	1 [Reference]	153 (21.3)	1 [Reference]
Annual family income						
<$25 000 (n = 176)	79 (45.1)	1.94 (1.53-2.46)	27 (17.5)	1.48 (0.98-2.21)	66 (36.5)	2.29 (1.64-3.19)
$25 000-$49 999 (n = 174)	64 (37.3)	1.41 (0.87-2.28)	25 (14.2)	1.15 (0.57-2.35)	47 (27.7)	1.52 (1.02-2.26)
$50 000-$74 999 (n = 159)	46 (30.0)	1.01 (0.69-1.49)	13 (7.3)	0.55 (0.29-1.03)	38 (25.5)	1.36 (0.87-2.13)
≥$75 000 (n = 599)	179 (29.8)	1 [Reference]	73 (12.6)	1 [Reference]	125 (20.1)	1 [Reference]

Abbreviations: ADHD, attention-deficit/hyperactivity disorder; GED, General Educational Development; NA, not applicable; OR, odds ratio.

^a^ Diagnoses were based on parent report. Data were from the first wave of the Adolescent Brain Cognitive Development Study. Results are weighted and unadjusted.

^b^ Includes either current ADHD medications or lifetime outpatient mental health care.

^c^ Analysis of race and ethnicity excludes “other, non-Hispanic” group.

Children receiving ADHD medications included a significantly higher percentage of boys (15.7% [121 of 826]) than girls (7.0% [28 of 108]), White children (14.8% [104 of 759]) than Black children (9.4% [22 of 206]), children of parents without a high school education (9 of 36 [32.2%]) than of parents with a bachelor’s degree or higher (11.5% [84 of 715]) (Table 1), and children with the combined subtype of ADHD (17.0% [83 of 505]) than with the inattentive subtype (9.5% [49 of 523]) (Table 3). Approximately 26.2% of children (301 of 1206) with parent-reported ADHD had ever received outpatient mental health care. Children receiving outpatient mental health care included a significantly higher percentage of children whose parents had a high school education (36.2% [29 of 90]) or some college (31.0% [109 of 364]) than a bachelor’s degree or higher (21.3% [153 of 715]), children with family incomes of less than $25 000 (36.5% [66 of 176]) or $25 000 to $49 999 (27.7% [47 of 174]) than $75 000 or more (20.1% [125 of 599]) (Table 1), and children with the combined subtype of ADHD (33.6% [166 of 505]) than with the predominantly inattentive subtype (20.0% [101 of 523]) or the hyperactive-impulsive subtype (22.4% [34 of 178]) ADHD (Table 3).

**Table 3.** Percentages of Children With Parent-Reported Current ADHD Who Received ADHD Treatment, Total and Stratified by ADHD Subtype and Selective Comorbid Mental Disorders^a^

Clinical correlate	Any treatment^b^		ADHD medications, % (95% CI)		Outpatient mental health care, % (95% CI)	
	No. (%)	OR (95% CI)	No. (%)	OR (95% CI)	No. (%)	OR (95% CI)
Total (n = 1206)	400 (34.8)	NA	149 (12.9)	NA	301 (26.2)	NA
ADHD subtype						
Inattentive (n = 523)	141 (27.8)	0.49 (0.39-0.62)	49 (9.5)	0.51 (0.35-0.75)	101 (20.0)	0.49 (0.38-0.65)
Hyperactive-impulsive (n = 178)	43 (27.3)	0.48 (0.33-0.70)	17 (10.6)	0.58 (0.31-1.07)	34 (22.4)	0.57 (0.39-0.83)
Combined (n = 505)	216 (44.0)	1 [Reference]	83 (17.0)	1 [Reference]	166 (33.6)	1 [Reference]
Comorbidity						
Disruptive behavior disorder (n = 405)^c^	182 (45.9)	2.08 (1.55-2.79)	64 (15.9)	1.47 (0.96-2.25)	146 (36.5)	2.19 (1.51-3.18)
No disruptive behavior disorder (n = 801)	218 (29.0)	1 [Reference]	85 (11.4)	1 [Reference]	155 (20.8)	1 [Reference]
Mood or anxiety disorder (n = 158)^d^	84 (52.9)	2.42 (1.53-3.81)	17 (13.2)	1.03 (0.51-2.06)	75 (44.7)	2.68 (1.86-3.85)
No mood or anxiety disorder (n = 1048)	316 (31.8)	1 [Reference]	132 (12.9)	1 [Reference]	226 (23.2)	1 [Reference]
OCD (n = 122)	64 (51.7)	2.21 (1.32-3.69)	22 (16.8)	1.42 (0.82-2.46)	51 (41.4)	2.19 (1.26-3.82)
No OCD (n = 1084)	336 (32.7)	1 [Reference]	127 (12.5)	1 [Reference]	250 (24.3)	1 [Reference]

Abbreviations: ADHD, attention-deficit/hyperactivity disorder; NA, not applicable; OCD, obsessive-compulsive disorder; OR, odds ratio.

^a^ Diagnoses were based on parent report. Data were from the first wave of the Adolescent Brain Cognitive Development Study. Results are weighted.

^b^ Includes either current ADHD medications or lifetime outpatient mental health care.

^c^ Includes oppositional defiant disorder or conduct disorder.

^d^ Includes major depressive disorder, persistent depressive disorder, and bipolar disorder; anxiety disorder includes panic disorder, separation anxiety disorder, social anxiety disorder, or generalized anxiety disorder.

Among those meeting the more stringent criteria of both parent-reported and teacher-reported ADHD (prevalence, 2.7% [n = 110]), 15 (12.3%) were currently receiving ADHD medications, 35 (33.7%) had ever received outpatient mental health care, and 44 (39.4%) had received either service.

In the parent-reported ADHD sample, the odds of any mental health care did not significantly vary by child sex or race and ethnicity or by parental marital status (Table 2). However, children with parent-reported ADHD whose parents had less education and whose families had lower incomes were more likely than those whose parents had more education and whose families had higher incomes to have received any treatment. Children from families in which a language other than English was spoken in the home were less likely to have received outpatient treatment. Associations between child sociodemographic characteristics and receipt of any treatment were slightly reduced after controlling for the potential confounders associated with ADHD subtype and the psychiatric comorbid conditions (Table 4). For example, the odds of any ADHD medication for boys compared with girls decreased from 2.46 (95% CI, 1.42-4.27) (Table 2) to 2.25 (95% CI, 1.27-4.00) (Table 4) after adjusting for ADHD subtype and comorbid conditions.

**Table 4.** Adjusted Odds of Any ADHD Treatment Among Children With Parent-Reported Current ADHD, Stratified by Sociodemographic Characteristics^a^

Characteristic	Any treatment		ADHD medications		Outpatient mental health care	
	OR (95% CI) adjusted for ADHD subtype	OR (95% CI)^b^	OR (95% CI) adjusted for ADHD subtype	OR (95% CI)^b^	OR (95% CI) adjusted for ADHD subtype	OR (95% CI)^b^
Sex, child						
Male	1.28 (0.93-1.77)	1.27 (0.92-1.75)	2.30 (1.31-4.04)	2.25 (1.27-4.00)	0.99 (0.67-1.46)	0.97 (0.65-1.45)
Female	1 [Reference]	1 [Reference]	1 [Reference]	1 [Reference]	1 [Reference]	1 [Reference]
Race and ethnicity, child						
Black, non-Hispanic	0.83 (0.58-1.18)	0.75 (0.51-1.10)	0.58 (0.35-0.96)	0.56 (0.34-0.92)	1.01 (0.67-1.54)	0.93 (0.59-1.46)
Hispanic	0.71 (0.48-1.07)	0.70 (0.49->1.00)	0.67 (0.42-1.06)	0.68 (0.42-1.10)	0.87 (0.47-1.60)	0.86 (0.49-1.49)
White, non-Hispanic	1 [Reference]	1 [Reference]	1 [Reference]	1 [Reference]	1 [Reference]	1 [Reference]
Language spoken at home						
English	1 [Reference]	1 [Reference]	1 [Reference]	1 [Reference]	1 [Reference]	1 [Reference]
Other	0.59 (0.32-1.12)	0.68 (0.37-1.23)	0.96 (0.45-2.04)	0.98 (0.50-1.93)	0.47 (0.24-0.93)	0.56 (0.28-1.14)
Marital status, parent						
Married or living with partner	1 [Reference]	1 [Reference]	1 [Reference]	1 [Reference]	1 [Reference]	1 [Reference]
Separated, divorced, or widowed	1.39 (0.86-2.24)	1.37 (0.82-2.29)	1.04 (0.68-1.60)	1.06 (0.68-1.66)	1.59 (0.90-2.82)	1.58 (0.85-2.92)
Never married	1.46 (0.77-2.75)	1.32 (0.73-2.38)	0.95 (0.50-1.82)	0.95 (0.50-1.80)	1.52 (0.82-2.84)	1.36 (0.75-2.47)
Highest parental education level						
<12th Grade	2.31 (0.83-6.38)	1.97 (0.70-5.56)	3.50 (1.60-7.65)	3.35 (1.42-7.91)	1.37 (0.67-2.78)	1.14 (0.59-2.22)
High school graduate or GED certification	1.53 (0.90-2.60)	1.25 (0.70-2.24)	1.28 (0.66-2.49)	1.21 (0.62-2.39)	1.94 (1.16-3.26)	1.60 (0.89-2.87)
Some college or Associate’s degree	1.54 (1.05-2.25)	1.38 (0.92-2.07)	1.08 (0.54-2.14)	1.06 (0.52-2.14)	1.63 (1.21-2.19)	1.46 (1.07-2.01)
Bachelor’s degree or higher	1 [Reference]	1 [Reference]	1 [Reference]	1 [Reference]	1 [Reference]	1 [Reference]
Annual family income						
<$25 000	1.78 (1.41-2.26)	1.56 (1.26-1.94)	1.36 (0.88-2.10)	1.39 (0.93-2.09)	2.12 (1.57-2.87)	1.82 (1.34-2.47)
$25 000-$49 999	1.30 (0.79-2.15)	1.21 (0.71-2.07)	1.07 (0.50-2.27)	1.07 (0.49-2.36)	1.41 (0.94-2.12)	1.30 (0.85-1.99)
$50 000-$74 999	0.95 (0.66-1.37)	0.86 (0.59-1.24)	0.51 (0.27-0.99)	0.52 (0.27-0.99)	1.29 (0.84-1.98)	1.14 (0.75-1.74)
≥$75 000	1 [Reference]	1 [Reference]	1 [Reference]	1 [Reference]	1 [Reference]	1 [Reference]

Abbreviations: ADHD, attention-deficit/hyperactivity disorder; GED, General Educational Development; OR, odds ratio.

^a^ Diagnoses were based on parent report. Data were from the first wave of the Adolescent Brain Cognitive Development Study. Results are weighted.

^b^ Adjusted for ADHD subtype, disruptive behavior disorder, mood disorder, obsessive-compulsive disorder, and anxiety disorder.

In unadjusted analyses, current ADHD medication use was significantly more common among boys than girls (OR, 2.46; 95% CI, 1.42-4.27) and among children whose parents did not have a high school education than children of parents with a bachelor’s degree (OR, 3.66; 95% CI, 1.72-7.77), but it was less common among Black than White children (OR, 0.60; 95% CI, 0.36-0.99) (Table 2). Lower parental educational level (parents without a high school education: OR, 1.46; 95% CI, 0.72-2.93) and lower family income (<$25 000: OR, 2.29; 95% CI, 1.64-3.19) were associated with higher odds of receiving outpatient treatment. The associations with treatment were marginally attenuated after controlling for ADHD subtype and psychiatric comorbidities (Table 4).

### Clinical Correlates of ADHD Treatment

Children with parented-reported inattentive ADHD were significantly less likely than those with the combined subtype to have received all 3 treatment outcomes (Table 3). Children with parent-reported hyperactive-impulsive ADHD were less likely than those with parent-reported combined ADHD to have received outpatient mental health care (OR, 0.57; 95% CI, 0.39-0.83). Compared with children without comorbid disruptive disorders, those with disruptive behavior disorders were more likely to have received outpatient mental health care (OR, 2.19; 95% CI, 1.51-3.18). Similar findings were noted for comorbid mood disorders, anxiety disorders, and obsessive-compulsive disorder. However, comorbid psychiatric disorders were not associated with increased odds of receiving ADHD medications.

### Correlates of Stimulants and Nonstimulant ADHD Medications

Approximately 8.0% of children (n = 97) with parent-reported ADHD were receiving stimulants, and 5.1% (n = 53) were receiving atomoxetine, guanfacine, or clonidine (Table 5). Stimulants were significantly more common among boys than girls (OR, 2.00; 95% CI, 1.14-3.50), although less common among Black than White children (OR, 0.44; 95% CI, 0.24-0.82). Nonstimulant ADHD medications were significantly more commonly prescribed to children whose parents had a high school diploma without attending college than to children whose parents had a bachelor’s degree or higher (OR, 2.79; 95% CI, 1.09-7.13). They were also more commonly prescribed to children with parent-reported combined ADHD than to children with parent-reported inattentive ADHD (OR, 2.74; 95% CI, 1.51-4.95), and to children with rather than without comorbid disruptive behavior disorders (OR, 2.80; 95% CI, 1.43-5.47).

**Table 5.** Sociodemographic Correlates of Stimulant and Other ADHD Medication Treatment of Children With Parent-Reported Current ADHD^a^

Sociodemographic characteristic	Any stimulant medication		Any nonstimulant ADHD medication	
	No. (%)	OR (95% CI)	No. (%)	OR (95% CI)
Total	97 (8.0)	NA	53 (5.1)	NA
Sex, child				
Male	77 (9.5)	2.00 (1.14-3.50)	45 (6.5)	3.27 (0.97-10.97)
Female	20 (5.0)	1 [Reference]	8 (2.1)	1 [Reference]
Race and ethnicity, child^b^				
Black, non-Hispanic	11 (4.4)	0.44 (0.24-0.82)	11 (5.0)	0.89 (0.48-1.65)
Hispanic	12 (7.0)	0.72 (0.40-1.29)	6 (3.5)	0.61 (0.22-1.69)
White, non-Hispanic	71 (9.5)	1 [Reference]	34 (5.6)	1 [Reference]
Language spoken at home				
English	88 (8.1)	1 [Reference]	47 (5.0)	1 [Reference]
Other	6 (11.4)	1.47 (0.66-3.23)	1 (0.8)	0.15 (0.02-1.44)
Marital status, parent				
Married or living with partner	70 (8.5)	1 [Reference]	34 (4.6)	1 [Reference]
Separated, divorced, or widowed	13 (7.4)	0.86 (0.45-1.67)	10 (6.6)	1.49 (0.68-3.25)
Never married	14 (7.4)	0.86 (0.36-2.06)	9 (5.5)	1.22 (0.63-2.37)
Highest parental educational level				
<12th Grade	6 (21.3)	3.22 (0.97-10.62)	3 (10.9)	3.16 (0.59-16.86)
High school graduate or GED certification	6 (5.5)	0.69 (0.32-1.49)	8 (9.7)	2.79 (1.09-7.13)
Some college or Associate’s degree	28 (7.7)	0.98 (0.52-1.86)	15 (5.4)	1.47 (0.67-3.25)
Bachelor’s degree or higher	57 (7.8)	1 [Reference]	27 (3.7)	1 [Reference]
Annual family income				
<$25 000	14 (8.4)	0.95 (0.41-2.24)	13 (9.1)	2.57 (0.84-7.88)
$25 000-$49 999	13 (7.8)	0.87 (0.38-2.01)	13 (7.3)	2.02 (0.88-4.62)
$50 000-$74 999	11 (5.9)	0.65 (0.32-1.34)	2 (1.3)	0.35 (0.08-1.49)
≥$75 000	52 (8.8)	1 [Reference]	21 (3.7)	1 [Reference]
ADHD subtype				
Inattentive	34 (6.6)	1 [Reference]	15 (2.9)	1 [Reference]
Impulsive or hyperactive	12 (6.7)	1.01 (0.45-2.28)	5 (3.9)	1.37 (0.40-4.72)
Combined	51 (9.8)	1.53 (0.94-2.51)	33 (7.6)	2.74 (1.51-4.95)
Psychiatric comorbidity				
Disruptive behavior disorder	37 (7.8)	0.95 (0.55-1.66)	28 (8.6)	2.80 (1.43-5.47)
No disruptive behavior disorder	60 (8.2)	1 [Reference]	25 (3.2)	1 [Reference]
Mood or anxiety disorder	9 (6.5)	0.77 (0.41-1.47)	8 (6.7)	1.42 (0.37-5.43)
No mood or anxiety disorder	88 (8.3)	1 [Reference]	45 (4.8)	1 [Reference]
OCD	13 (9.7)	1.27 (0.58-2.78)	9 (7.1)	1.50 (0.92-2.43)
No OCD	84 (7.8)	1 [Reference]	44 (4.8)	1 [Reference]

Abbreviations: ADHD, attention-deficit/hyperactivity disorder; GED, General Educational Development; NA, not applicable; OCD, obsessive-compulsive disorder; OR, odds ratio.

^a^ ADHD based on parent report. Data were from the first wave of the Adolescent Brain Cognitive Development Study. Percentages are weighted. A total of 0.2% of patients received stimulant and a nonstimulant ADHD medication.

^b^ Analysis of race and ethnicity excludes “other, non-Hispanic” group.

## Discussion

In this cohort of children with parent-reported ADHD, 26.2% had ever received outpatient mental health care, 12.9% were currently receiving ADHD medications, and 34.8% had received either treatment. Contrary to our expectations, children whose parents had higher incomes and a higher educational level tended to be less likely than those whose parents had lower incomes and a lower educational level to have received outpatient mental health care. Medication treatment for children with parent-reported ADHD was also more common among children whose parents had a lower rather than higher educational level. Use of ADHD medications was more common among boys than girls, White children than Black children, and children with combined rather than inattentive ADHD.

Low treatment rates among children with parent-reported ADHD suggest a need to increase mental health service availability; enhance knowledge of ADHD symptoms among parents, teachers, and primary care clinicians; and develop accessible care pathways.^38^ Because most US children receive an annual well-child visit,^39^ opportunities exist to improve ADHD detection in primary care. However, because no screening tool has sufficient accuracy for stand-alone use,^40^ primary care clinicians should consider multiple informants when evaluating children for ADHD. Local shortages of child mental health clinicians also hinder care access in many communities.^41^

These ADHD treatment rates contrast with CDC reports that most children with ADHD receive treatment.^42,43,44^ This discrepancy is likely associated with ascertainment bias. Unlike the current analysis, the CDC reports were based on parent-reported treatment of children who had received a clinical ADHD diagnosis. By contrast, the current treatment rates in our study more closely align with earlier epidemiologic studies.^45^

In keeping with prior research,^2,14,15,16,17^ boys were more likely than girls to receive ADHD medications. A common explanation for this sex-based difference is that, because boys tend to have more disruptive clinical presentations than girls, they have a higher likelihood of referral for evaluation.^46^ However, we found that boys compared with girls had more than twice the odds of receiving ADHD medications after adjustment for ADHD subtype, comorbid disruptive behavior disorders, and other common psychiatric comorbid conditions. Other factors, such as diagnostic biases favoring boys vs girls,^47^ greater prevalence of educational problems in boys compared with girls,^14^ or higher levels of physical aggression in boys than in girls with ADHD^48^ might be associated with greater ADHD medication use among boys than girls.

Children with parent-reported ADHD from families with the highest income level were less likely than those from families with the lowest income level to receive outpatient mental health care. It is unclear what inhibited highly resourced families from seeking treatment. Compared with families with lower income, those with higher income might more frequently rely on nontraditional strategies, such as tutoring, exercise, sports programs, and healthy eating,^49^ or have greater apprehension concerning the stigma of receiving treatment or the long-term effects of stimulant therapy.^50^

Black children compared with White children with parent-reported ADHD were significantly less likely to receive ADHD medications. This finding is broadly consistent with prior research.^51^ Black children were specifically less likely than White children to receive stimulants, while this difference was less pronounced for nonstimulants. Because nonstimulant medications tend to yield smaller therapeutic effects than stimulants,^52,53^ all parents should be provided with relevant clinical information to make informed ADHD medication choices. Prescriber implicit bias might be associated with disparities in stimulant prescribing.^54^ Strong population-based racial and ethnic gradients exist in prescriptions for stimulants and other controlled substances, with the highest rates in majority-White areas.^55^ As a result of structural racism,^56^ Black parents’ perspectives might further influence ADHD management decisions through mistrust in clinicians and concerns over safety and efficacy of stimulants.^57^ Physician efforts to recognize and manage their own implicit biases,^58^ together with patient-centered clinical approaches that promote shared decision-making, including open explorations of Black parents’ knowledge, attitudes, and beliefs concerning ADHD and its management, might help reduce these treatment disparities.

Children whose parents had the lowest educational level were most likely to receive ADHD medications. Earlier research has reported an association between low parental educational level and ADHD medication prescription.^59,60^ In a discrete choice study of parents of young children with medication-naive ADHD, medication-avoidant parents had a significantly higher educational level than more outcome-oriented parents who favored the use of ADHD medication.^61^ As a group, parents with a lower educational level may be more focused on improving their child’s functioning than parents with a higher educational level who may be more concerned with medication-associated risks and might therefore be more motivated to implement parent-based interventions.

Children with parent-reported combined ADHD tended to be more likely than those with either inattentive or hyperactive-impulsive ADHD to receive ADHD medications. This pattern contrasts with a previous report that hyperactivity is specifically associated with ADHD medication treatment,^26^ but it supports the hypothesis that children with more severe or complex presentations are more likely to receive ADHD medications. Common psychiatric comorbid conditions were not significantly associated with increased likelihood of ADHD medication use. This finding suggests that community ADHD medication prescribing practices are appropriately and specifically focused on ADHD symptoms. In evaluating the finding that 57.0% of all children who received ADHD medications did not have parent-reported ADHD, it is important to consider the possibility of symptom response.

Among children with parent-reported ADHD, nonstimulant ADHD medications were significantly more commonly prescribed to children with combined ADHD than inattentive ADHD and were more commonly prescribed to those with than without comorbid disruptive behavior disorders. Although there is some evidence to support the use of guanfacine and atomoxetine for disruptive behavior disorders, psychostimulants have more evidence for effectiveness, and there is little evidence of the effectiveness of clonidine.^62^

### Limitations

This study has several limitations. First, although the ABCD Study involved multistage probability sampling of schools and survey weights were applied, the study did not involve nationally representative sampling,^30^ and stimulant prescriptions have demonstrated substantial regional variation.^23^ Second, treatment and medication assessments were based on parent reports without confirmatory administrative or prescription data, and no information was available concerning medication dose, duration, or prescriber. Because many children with ADHD stop and start stimulants,^63^ measuring medication use in the past 2 weeks will miss children who recently stopped medications. Third, information was also not available concerning the clinical focus of the lifetime child mental health care, whether it occurred before or after the onset of ADHD, or whether it included evidence-based treatments, such as parent training in child behavioral management^62^ for ADHD. Parents may also vary in how they define what constitutes outpatient mental health care. Fourth, the study was limited to a narrow age range, and treatment patterns vary by patient age. Fifth, eligibility criteria for the ABCD Study, which include English fluency, gestational age, birth weight, major medical conditions, and other factors, as well as imperfections in the survey weights, limit the generalizability of the study findings. Sixth, parent-reported ADHD may have excluded children who, due to active interventions, did not meet ADHD criteria.

## Conclusions

In this cross-sectional study of children with parent-reported ADHD, 26.2% had ever received outpatient mental health care, and 15.7% of boys and 7.0% of girls were currently receiving ADHD medications. These findings contrast with the view that most children with ADHD receive treatment for their symptoms.^42,43,44^ Contrary to our expectations, children with ADHD from families with a lower educational level and lower incomes tended to be more likely than those with a higher educational level and higher incomes to have received outpatient mental health treatment. These patterns suggest that attitudinal rather than socioeconomic factors often impede the flow of children with ADHD into treatment.

It would be naive to assume that all children with parent-reported ADHD need or would benefit from treatment. Parent reports from research diagnostic interviews do not substitute for a thorough clinical assessment involving a patient history, clinical child interview and observation, physical examination, teacher reports, and validated rating scales. Nevertheless, the findings provide a sense of the scale and distribution of children with parent-reported ADHD not receiving treatment. As such, they highlight the importance of developing strategies to increase clinical recognition of children with ADHD, especially girls, and increasing access to acceptable treatments.

## Notes

Supplement 1. **eTable.** Selected Sociodemographic and Physical Characteristics of the Total ABCD Sample and Propensity-Based Population Reweighted Sample With Teacher Responses

Supplement 2.
Data Sharing Statement
