# Supplementary material for: Treatment of US Children With Attention-Deficit/Hyperactivity Disorder in the Adolescent Brain Cognitive Development Study
Source: JAMA Netw Open. 2023 Apr 28;6(4):e2310999. doi: 10.1001/jamanetworkopen.2023.10999 (PMC10148191; doi:10.1001/jamanetworkopen.2023.10999)
Supplement: Supplement 1. — eTable. Selected Sociodemographic and Physical Characteristics of the Total ABCD Sample and Propensity-Based Population Reweighted Sample With Teacher Responses [file jamanetwopen-e2310999-s001.pdf]

Supplementary Online Content

Olfson M, Wall MM, Wang S, Laje G, Blanco C. Treatment of US children with attention-deficit/hyperactivity disorder in the Adolescent Brain Cognitive Development Study. *JAMA Netw Open.* 2023;6(4):e2310999. doi:10.1001/jamanetworkopen.2023.10999

eTable. Selected Sociodemographic and Physical Characteristics of the Total ABCD Sample and Propensity-Based Population Reweighted Sample With Teacher Responses

This supplementary material has been provided by the authors to give readers additional information about their work.

© 2023 Olfson M et al. *JAMA Network Open.*

**eTable.** Selected Sociodemographic and Physical Characteristics of the Total ABCD Sample and Propensity-Based Population Reweighted Sample With Teacher Responses					
Characteristics	Total Sample (N=11,874) %[A]	Sample with Teacher Responses (N=4,594) %	SMD Total Sample – Sample with Teacher Responses	Reweighted Sample with Teacher Responses (N=4,594) %	SMD Total Sample – Reweighted Sample with Teacher Responses
**Sex,** child					
Male	51.2	50.4	0.0160	50.9	0.0068
Female	48.8	49.6	-0.0160	49.2	-0.0068
**Race/Ethnicity,** child					
Hispanic	24.2	17.0	0.1776	21.4	0.0650
White, non-Hispanic	55.8	65.8	-0.2061	58.9	-0.0634
Black, non-Hispanic	13.8	11.2	0.0793	13.4	0.0125
Other, non-Hispanic	6.3	6.0	0.0098	6.3	-0.0008
**Nativity**, child					
United States	96.2	97.3	-0.0625	96.6	-0.0215
Other	3.8	2.7	0.0625	3.4	0.0215
**Marital status**, parent					
Married or Living with Partner	67.2	72.1	-0.1074	69.3	-0.0463
Separated or Divorced	17.2	16.1	0.0270	16.9	0.0074
Never married	14.5	11.0	0.1047	13.0	0.0429
Widowed	1.1	0.7	0.0449	0.8	0.0405
**Education,** highest parent					
<12th grade	6.1	3.5	0.1224	5.1	0.0430
High school graduate/GED	11.2	8.8	0.0821	10.0	0.0397
Some college/Associates Degree	29.7	27.9	0.0409	29.8	-0.0025
Bachelor’s Degree or Higher	52.9	59.8	-0.1400	55.0	-0.0422
**Annual family income**					
<$25,000	19.0	14.6	0.1189	18.5	0.0120
$25,000-$49,999	20.1	18.2	0.0500	19.9	0.0059
$50,000-$74,999	17.4	18.5	-0.0282	17.7	-0.0074
$75,000+	43.4	48.8	-0.1068	43.8	-0.0085
**Pubertal stage**					
Prepubertal	48.4	50.4	-0.0399	48.6	-0.0044
Early	24.5	23.9	0.0150	24.2	0.0062
Middle or Later	27.1	25.7	0.0308	27.1	-0.0010
**Weight status**					
Healthy weight	62.5	66.4	-0.0813	64.5	-0.0417
Underweight	4.0	3.6	0.0197	3.5	0.0255
Overweight	17.0	15.4	0.0420	16.0	0.0249
Obese	16.5	14.6	0.0543	15.9	0.0162
**Maternal age at child’s birth, years**[B]					
≤19 years	5.9	4.6	0.0588	5.4	0.0213
20-34 years	73.6	74.5	-0.0197	74.8	-0.0265
≥35 years	20.5	20.9	-0.0111	19.8	0.0166
**Gestation period**					
Term	86.3	86.5	-0.0075	86.8	-0.0168
Pre-term (<37 weeks)	13.7	13.5	0.0075	13.2	0.0168
**Region**					
Northeast	22.6	20.8	0.0442	22.7	-0.0023
Midwest	23.6	28.7	-0.1169	24.3	-0.0162
South	32.2	29.6	0.0561	31.4	0.0171
West	21.6	20.9	0.0175	21.6	-0.0003
Data from ABCD study. SMD denotes standardized mean difference. [A]Percentages are weighted. [B]Limited to children in which respondent in biological mother, N=10,056.					

© 2023 Olfson M et al. *JAMA Network Open.*
