# Supplementary material for: Treatment of US Children With Attention-Deficit/Hyperactivity Disorder in the Adolescent Brain Cognitive Development Study
Source: JAMA Netw Open. 2023 Apr 28;6(4):e2310999. doi: 10.1001/jamanetworkopen.2023.10999 (PMC10148191; doi:10.1001/jamanetworkopen.2023.10999)
Supplement: Supplement 2. — Data Sharing Statement [file jamanetwopen-e2310999-s002.pdf]

Data Sharing Statement

Olfson. Treatment of US Children With Attention-Deficit/Hyperactivity Disorder in the Adolescent Brain Cognitive Development Study. *JAMA Netw Open*. Published April 28, 2023. doi:10.1001/jamanetworkopen.2023.10999

Data
Data available: Yes
Data types: Deidentified participant data, Data dictionary
How to access data: : https://abcdstudy.org/wp-content/uploads/2021/11/Data_release_schedule_update2021.pdf?swcfpc=1
When available: beginning date: 01-15-2022

Supporting Documents
Document types: Statistical/analytic code
How to access documents: The code will be made available to the public via GitHub: https://github.com/Olfsonm/MHCODE.git
When available: With publication

Additional Information
Who can access the data: Anyone requesting the data from NIDA.
Types of analyses: Research purposes.
Mechanisms of data availability: Without investigator support
Any additional restrictions: None
